# Relation of Homœopathy to Natural Science

**Published:** 1889-12

**Authors:** 


Relation of Homœopathy to Natural Science. By E. B. Atkins, M. D. Pp. 195. Price, $1.00. A. L. Chatterton & Co., New York.

The title of Dr. Atkins' work clearly explains the scope and purpose of the volume, *i. e.*, to show the claims of Homœopathy to the title of "the science of therapeutics." The volume is very readable and will well repay perusal. In regard to the object of the volume, we may say that although much scientific proof may be adduced in favor of the truth of the homœopathic law, some data are yet lacking, owing to Homœopathy being in a measure in advance of physical science.

